# Long non-coding RNAs as novel expression signatures modulate DNA damage and repair in cadmium toxicology

**DOI:** 10.1038/srep15293

**Published:** 2015-10-16

**Authors:** Zhiheng Zhou, Haibai Liu, Caixia Wang, Qian Lu, Qinhai Huang, Chanjiao Zheng, Yixiong Lei

**Affiliations:** 1 School of Public Health, Guangzhou Medical University, Guangzhou 510182, People’s Republic of China; 2 Department of Internal Medicine of Guangzhou First People’s Hospital, Guangzhou Medical University, Guangzhou 510180, P.R. China; 3 Shenzhen Longgang District Center for Disease Control & Prevention, Shenzhen 518108, P.R. China

## Abstract

Increasing evidence suggests that long non-coding RNAs (lncRNAs) are involved in a variety of physiological and pathophysiological processes. Our study was to investigate whether lncRNAs as novel expression signatures are able to modulate DNA damage and repair in cadmium(Cd) toxicity. There were aberrant expression profiles of lncRNAs in 35th Cd-induced cells as compared to untreated 16HBE cells. siRNA-mediated knockdown of ENST00000414355 inhibited the growth of DNA-damaged cells and decreased the expressions of DNA-damage related genes (ATM, ATR and ATRIP), while increased the expressions of DNA-repair related genes (DDB1, DDB2, OGG1, ERCC1, MSH2, RAD50, XRCC1 and BARD1). Cadmium increased ENST00000414355 expression in the lung of Cd-exposed rats in a dose-dependent manner. A significant positive correlation was observed between blood ENST00000414355 expression and urinary/blood Cd concentrations, and there were significant correlations of lncRNA-ENST00000414355 expression with the expressions of target genes in the lung of Cd-exposed rats and the blood of Cd exposed workers. These results indicate that some lncRNAs are aberrantly expressed in Cd-treated 16HBE cells. lncRNA-ENST00000414355 may serve as a signature for DNA damage and repair related to the epigenetic mechanisms underlying the cadmium toxicity and become a novel biomarker of cadmium toxicity.

Genome-wide transcriptome studies have revealed that the mammalian genome encodes a novel class of regulatory genes known as long non-coding RNAs (lncRNAs), which have >200 nulectides in length but lack obvious open reading frames. It is believed that the genome encodes at least as many lncRNAs as known protein-coding genes^1^,^2^. Thousands of lncRNAs have been found to be evolutionarily conserved^3^,^4^ and exhibit expression patterns correlating with various cellular processes^3^,^4^,^5^,^6^,^7^,^8^,^9^. It is now considered that these lncRNAs represent a feature of normal cellular networks. Specifically, increasing evidence suggests that lncRNAs play a critical role in the regulation of diverse cellular processes such as stem cell pluripotency, development, cell growth and apoptosis^3^,^4^,^5^,^6^,^7^,^8^,^9^. Given their abundance and regulatory potential, it is likely that some lncRNAs are involved in tumor initiation and progression. In support of this notion, several lncRNAs are found to be aberrantly expressed in various human cancers, with potential roles in both oncogenic and tumor suppressive pathways^10^,^11^,^12^,^13^,^14^. Furthermore, lncRNAs have been shown to play active roles in modulating the cancer epigenome^15^.

Recent studies suggest a number of modes of action for lncRNAs^16^, most notably the regulation of epigenetic marks and gene expression^6^,^17^,^18^,^19^. In addition, lncRNAs may function as decoy, scaffold and guide molecules^1^. Some lncRNAs act in cis to regulate the transcription of nearby gene(s)^20^,^21^, while others act in trans to repress their transcription^22^.

Cadmium(Cd) is a heavy metal with widespread industrial application. However, it is toxic, and occupational and environmental exposure to it harms human health^23^,^24^,^25^. Experimental and epidemiological studies have shown that cadmium and its compounds are carcinogenic to animals and humans^26^,^27^,^28^. Cadmium and its compounds were classified as human carcinogens in 1993 by the International Agency for Research on Cancer^29^. Although some of the molecules involved in Cd tolerance have been identified, the regulatory mechanisms involved are still largely unknown. Reports suggest that the respiratory system is an important target organ for cadmium-induced toxicity and carcinogenicity, and Cd may lead to aberrant DNA methylation and different microRNAs expression profiles, which play important roles in modulating the expression of many genes^30^. To date, no study has been conducted to investigate the role of lncRNA in the cadmium-induced toxicity and carcinogenicity.

We previously established a model of morphological cell transformation with Cadmium chloride (CdCl_2_) in human bronchial epithelial cells (16HBE)^31^ and a Cd exposure rat model^32^. These models are helpful to examine the molecular events occurring during Cd toxicity and carcinogenesis. Our previous results showed that Cd increased cell apoptosis and DNA damage, and decreased DNA repair capacity. In the present study, we hypothesized that there were aberrant lncRNA expression in Cd treated cells, and the inactivation of DNA damage and repair pathways resulting from abnormal lncRNA expression profiles might play an important role in the Cd induced toxicity. To test this hypothesis, the lncRNA and mRNA expression profiles were detected in 35^th^ Cd-induced 16HBE cells and untreated 16HBE cells by microarray, and lncRNAs were found to be novel expression signatures modulating DNA damage and repair in Cd-induced malignant transformation of 16HBE cells, Cd-exposed rats and Cd-exposed workers.

## Results

### LncRNA expression profiles

**Figure 1:**
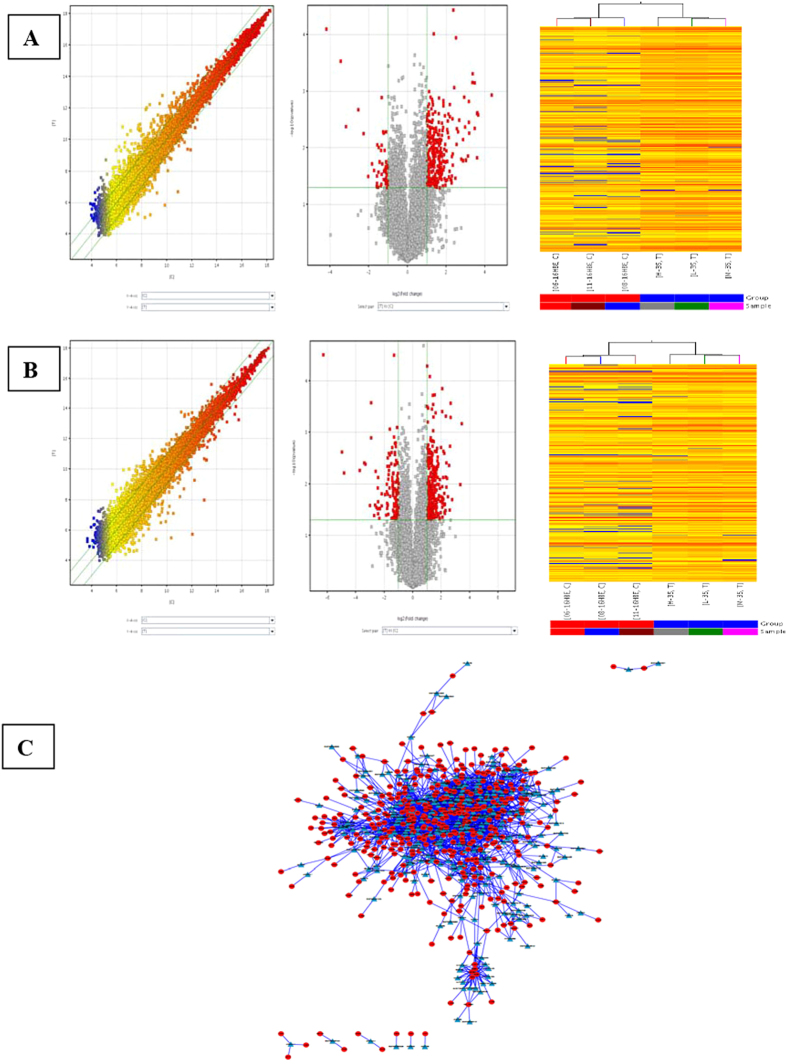
LncRNA and mRNA expression profiles and co-expression network in Cd-induced 35^th^ cells (T) as compared to untreated 16HBE cells (C). Hierarchical clustering was performed to show the distinguishable lncRNAs and mRNAs expression patterns in Cd-induced 35^th^ cells (T) as compared to untreated 16HBE cells. (**A**) Differentially expressed lncRNAs and (**B**) differentially expressed mRNAs were detected. The A and B left panels showed the scatter-plot for T vs C. The A and B middle panels showed the volcano plot which was constructed using 2.0-fold up and down change values and p-values of 0.05. Red point in the plot represented the differentially expressed lncRNAs and mRNAs with statistical significance. The A and B right panels showed Hierarchical clustering for differentially expressed lncRNAs and mRNAs in T vs C. “Red” indicated high relative expression, and “blue” indicated low relative expression. (**C**) CNC network was constructed based on the correlation analysis between differentially expressed lncRNAs and mRNAs in Cd-induced 35^th^ 16HBE cells as compared to untreated 16HBE cells. Triangle: lncRNA; circle: mRNA.

Based on the lncRNA expression profiles (Table S1), differentially expressed lncRNAs were found between Cd-induced 35^th^ cells (T) and untreated 16HBE cells (N). The lncRNA expression profiles were shown by calculating the log-fold change (T/N). With abundant and varied probes (33,045 lncRNAs) in the microarray, the number of detectable lncRNAs was 21409. Of them, there were 369 lncRNAs with up-regulated expression and 90 lncRNAs with down-regulated expression (≥2.0 fold-change, P < 0.05) in Cd-induced 35^th^ cells when compared with untreated 16HBE cells. Our results showed that the number of up-regulated lncRNAs was larger than that of down-regulated ones (Fig. 1A, Table S2).

### mRNA expression profiles

With abundant and varied probes (30215 mRNAs) in the microarray, the number of detectable mRNAs was 18185 (Table S3). Of them, there were 366 mRNAs with up-regulated expression and 132 mRNAs with down-regulated expression (≥2.0 fold-change, P < 0.05) in Cd-induced 35^th^ cells when compared with untreated 16HBE cells (Fig. 1B, Table S4).

### Gene ontology (GO) and pathway analysis

GO analysis showed that the genes with aberrant mRNA expression mainly took part in following biological processes (Fig. S1, Table S5): primary metabolism, cell metabolism, cell cycle progression, DNA damage and repair, biological cycle, etc. Pathway analysis of differentially expressed mRNAs (Fig. S2, Table S6) showed they were involved in the cell cycle, P53 signaling pathway, prostate cancer, thyroid cancer, and endometrial cancer, Wnt signaling pathway, glioma, bladder cancer, pancreatic cancer and axon guidance. These results support the idea that Cd-induced malignant transformation is related to DNA damage, DNA repair and biological cycle.

### Construction of coding-non-coding gene co-expression network

A coding-non-coding gene co-expression network (CNC network) was constructed based on the correlation analysis between differentially expressed lncRNAs and mRNAs. lncRNAs and mRNAs which had Pearson’s correlation coefficients of no less than 0.99 were used to construct the network. In total, 322 lncRNAs and 468 mRNAs were included in the co-expression network. In addition, 2,988 network nodes were associated with 15,1018 network pairs of co-expressing lncRNAs and mRNAs, and most of these pairs showed a positive correlation. The CNC networks indicated that one mRNA was correlated with one to ten lncRNAs and so were the lncRNAs. The CNC networks in Fig. 1C implicate that the inter-regulation of lncRNA and mRNA is involved in Cd-induced malignant transformation of human bronchial epithelial cells.

### Validation by real-time quantitative PCR

**Figure 2:**
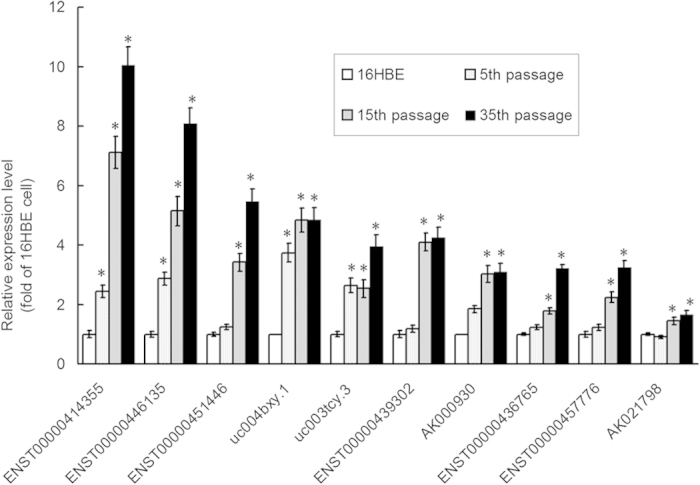
Validation of miroarray findings by qPCR. The expression of 10 lncRNAs were validated in untreated 16HBE cells, Cd-induced 5^th^, 15^th^ treated cells, and 35^th^ passage transformed cells by qPCR and normalized to that of β-actin. The fold change in expression was expressed as the expression in experiment group normalized to that in untreated 16HBE cells. Data are expressed as mean ± SD. *P < 0.05 vs untreated 16HBE cells (one-way ANOVA).

To validate the findings from microarray assay, the expression of 10 lncRNAs correlated with antecedent mRNAs among over-expressing lncRNAs, was determined by quantitative real-time polymerase chain reaction (qRT-PCR). Results showed that the expression of 10 lncRNAs in human bronchial epithelial cells with Cd-induced malignant transformation was up-regulated when compared with matched 16HBE cells (P < 0.05; Fig. 2), which was consistent with the findings from microarray assay. These results indicate that some lncRNAs are aberrantly expressed in human bronchial epithelial cells with Cd-induced malignant transformation.

### Bioinformatics analysis of lncRNA-ENST00000414355

**Figure 3:**
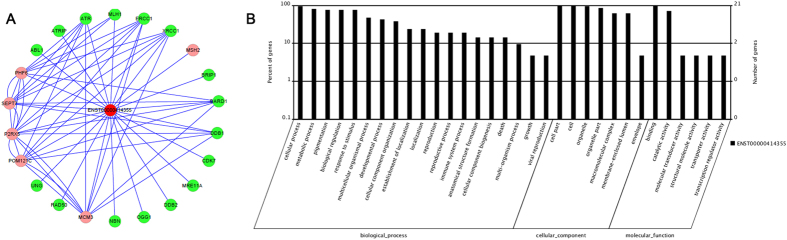
Bioinformatics analysis of lncRNA-ENST00000414355. (**A**) Co-expression network of LncRNA-ENST00000414355 and mRNA was constructed with cytoscopesoftware (http://www.cytoscape.org/) based on the correlation analysis between LncRNA-ENST00000414355 and differentially expressed mRNAs in Cd-induced 35^th^ 16HBE cells as compared to untreated 16HBE cells. (**B**) GO and signaling pathway analysis of LncRNA-ENST00000414355. Pathway analysis was predominantly based on the KEGG database, and the mRNAs were annotated and classified according to the GO database.

According to the GO analysis and pathway analysis of differentially expressed lncRNAs/mRNAs, lncRNA-ENST00000414355 was selected for further analysis. As shown in the coding-noncoding co-expression network (Fig. 3A), ENST00000414355 and their associated mRNAs were identified, with most of the pairs showing a positive correlation. The neighbor gene function of upregulated ENST00000414355 mainly involved the following pathway to the target genes: DNA damage and repair, biological cycle, metabolism, molecular transducer activity, cell cycle progression, etc (Fig. 3B, Table S7). There were 22 potential target mRNAs regulated by ENST00000414355, and most of the target mRNAs have been reported to be related to cancers.

### Silencing of ENST00000414355 decreased DNA damage in human bronchial epithelial cells with Cd-induced malignant transformation

During the Cd-induced malignant transformation of 16HBE cells, the tail lengths of the DNA comets were significantly longer than those in the untransformed 16HBE cells (P < 0.05), and the tail lengths of the DNA comets in Cd-treated 35^th^ passage cells were significantly longer than those in siRNA ENST00000414355 transfected cells (P < 0.05). The Cd-induced DNA damage rates in 5^th^, 15^th^, and 35^th^ passage cells were 10.45%, 22.00% and 46.75%, respectively, as compared to untreated 16HBE cells (4.75%; P < 0.05). However, the DNA damage rate in Cd-induced 35^th^ passage cell after siRNA ENST00000414355 transfection were 23.98%, which was significantly lower than that in negative control group (P < 0.05) (Table 1). These results suggest that siRNA ENST00000414355 inhibits the growth of DNA-damaged cells during the Cd-induced malignant transformation of 16HBE cells.

### Silencing of ENST00000414355 increased/decreased the expression of DNA damage and repair related genes in 16HBE cells with Cd-induced malignant transformation

**Figure 4:**
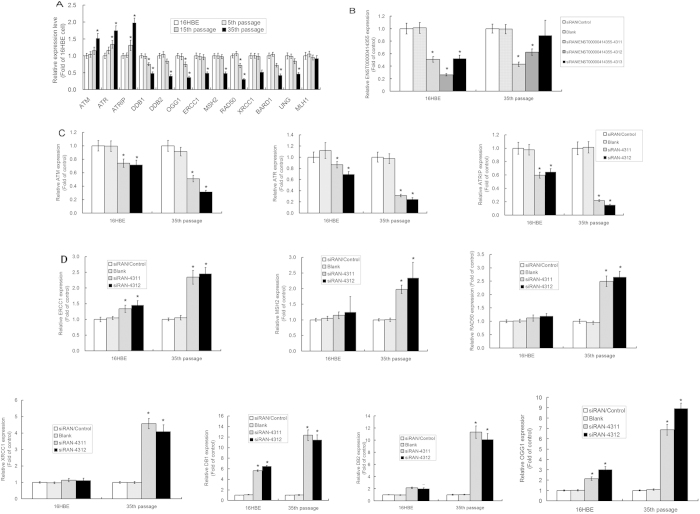
mRNA expression of DNA damage and repair related genes in 16HBE cells with Cd induced malignant transformation and siRNA/lncRNA-ENST00000414355 transfected cells. (**A**) mRNA expression of DNA damage and repair related genes in 16HBE cells with Cd induced malignant transformation. The gene expression was validated in untreated controls 16HBE cells, Cd-induced 5^th^, 15^th^ treated cells, 35^th^ passage transformed cells by qPCR and normalized to that of β-actin. The fold change in expression of experiment group was normalzied to that of untreated control 16HBE cells. Data are expressed as mean ± SD. *P < 0.05 vs untreated 16HBE (one-way ANOVA). (**B**) Untreated control 16HBE cells and Cd-transformed 35^th^ passage cells were treated with lncRNA-ENST00000414355-siRNA, and the lncRNA-ENST00000414355 expression was detected after 72 h by qPCR. *P < 0.05 vs control cells (one-way ANOVA). (**C**) Untreated 16HBE cells and Cd-transformed 35^th^ passage cells were independently treated with lncRNA-ENST00000414355-siRNA531 and siRNA532, and the mRNA expression of DNA damage related genes was detected after 48 h by qPCR. (**D**) Untreated 16HBE cells and Cd-transformed 35^th^ passage cells were independently treated with lncRNA-ENST00000414355-siRNA531 and siRNA532, and the mRNA expression of DNA repair related genes was detected after 72 h by qPCR. *P < 0.05 vs control cells (one-way ANOVA).

The mRNA expression of genes related to DNA damage and repair was detected by real-time PCR. Results showed the mRNA expression of ATM, ATR and ATRIP progressively increased, but that of DDB1, DDB2, OGG1, ERCC1, MSH2, RAD50 and XRCC1 progressively reduced during the Cd-induced malignant transformation in human bronchial epithelial cells. Furthermore, the expression of these genes in 35^th^ passage-transformed cells was significantly different from that in untreated 16HBE cells (P < 0.05) (Fig. 4A). Transfection with siRNA ENST00000414355-4311 and siRNA ENST00000414355-4312 in Cd-treated 35^th^ passage transformed cells (Fig. 4B) resulted in a significant decrease in the mRNA expression of DNA damage related genes ATM, ATR and ATRIP, and a marked increase in the mRNA expression of DNA repair related genes which are a negative regulator of DNA repair signaling pathway (Fig. 4C,D). These findings suggest that siRNA ENST00000414355 activates DNA damage and repair signaling pathway.

### Bioinformatics analysis of ENST00000414355 and DDB1, DDB2 and OGG1

In order to further explore the mechanisms that ENST00000414355 modulated the expression of DNA repair related genes DDB1, DDB2 and OGG1, the RNA-protein interaction of lncRNA and corresponding TFS was analyzed based on ChIP-Seq database. Results showed that there were highly enriched region between ENST00000414355 and DDB1, DDB2 and OGG1 by comparing their promoter regions. The prediction of TFBS (trascription factor binding sites) and further catRAPID analysis indicated that polymerase 2 (Pol2) was a strong RNA-protein interaction between ENST00000414355 and DDB1, DDB2 and OGG1, which may be a possible mechanism underlying the ENST00000414355 modulating DNA repair related genes DDB1, DDB2 and OGG1 according to the natural antisense transcripts (NAT) theory.

### ENST00000414355 expression increased in Cd exposed rats

The expression of ENST00000414355 in Cd-exposed rats was confirmed by qPCR. The expression of ENST00000414355 in the lung of low dose, mid-dose and high dose Cd exposed rats was 2.132 ± 0.187-fold, 4.421 ± 0.411-fold and 8.674 ± 0.739-fold, respectively, as compared to that in control rats. Significantly up-regulated ENST00000414355 expression was found in the lung of Cd-exposed rats (P < 0.05). Additionally, Cd increased ENST00000414355 expression in the lung in a dose dependent manner (*P* < 0.05).

### ENST00000414355 expression was correlated with target gene expression in Cd exposed rats

As shown in Table 2, a significant positive correlation of ENST00000414355 expression with the expression of ATM, ATR and ATRIP was observed, while there was a significant negative correlation between ENST00000414355 expression and expression of DDB1, DDB2, OGG1, ERCC1, MSH2, RAD50 and XRCC1 in Cd exposed rats. These findings indicate that ENST00000414355 expression correlates well with the expression of its target genes in Cd exposed rats.

### Health status of subjects exposed to Cd

The subjects (median age, 31 years) were directly or indirectly exposed to Cd for less than 2 years with no history of exposures to other toxins. Only non-smokers were included in the present study. Urine Cd concentration normalized to the urine creatinine (Cr) showed a positive skewness distribution. The median, maximum and minimum urine Cd concentrations were 1.61, 113.86 and 0.31 μg/g.Cr, respectively. The 25 percentile and 75 percentile of urine Cd concentrations were 0.69 and 9.54 μg/g.Cr, respectively. According to urine Cd concentration, subjects were categorized into three groups: 0–2 μg/g Cr, 2–5 μg/g Cr and >5 μg/g Cr (Table 3). The age, gender and duration of employment were comparable among three groups, suggesting that our results were not confounded by these factors.

### ENST00000414355 expression was correlated with Cd exposure in Cd-exposed workers

**Figure 5:**
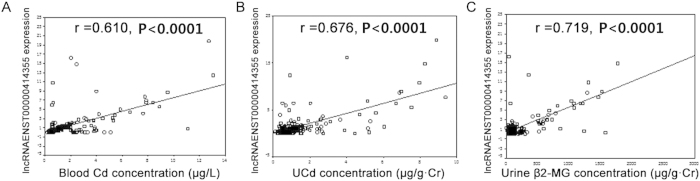
Correlation analysis between lncRNA-ENST00000414355 expression and Cd concentration in Cd-exposed workers. Correlation analysis between lncRNA-ENST00000414355 expression and blood Cd concentration (**A**), urine Cd concentration (**B**) and Urine β2-MG concentration (**C**). Blood lncRNA-ENST00000414355 expression was calculated by the ratio of its expression to that of β-actin. The urine cadmium concentration was normalized by urine creatinine (μg/L.Cr) and urine β2-MG (μg/g.Cr). The linear relationship was analyzed by Pearson correlation analysis.

In order to evaluate whether ENST00000414355 serves as a biomarker of Cd exposure, the expression of ENST00000414355 in the blood of Cd-exposed workers was detected by quantitative real-time PCR. According to the urine Cd concentration and blood Cd concentration, these works were divided into three groups (Table 3). The blood ENST00000414355 expression increased with the increase in urine Cd concentration and blood Cd concentration. The ENST00000414355 expression was significantly higher in workers with urine Cd concentration at 2–5 μg/g Cr and >5 μg/g Cr (2.647-fold and 7.1136-fold, respectively), when compared with control group (urine Cd concentration: 0–2 μg/g Cr) (P < 0.05). A similar finding was identified in blood ENST00000414355 expression in workers with different blood Cd concentrations (2.023-fold and 6.126-fold, respectively) when compared with control group (0–2 μg/l) (P < 0.05). There was a significant positive correlation between ENST00000414355 expression and blood Cd concentration (r = 0.610, P < 0.0001), urine Cd concentration (r = 0.676, P < 0.0001) and urine β_2_-MG concentration (r = 0.719, P < 0.0001) (Fig. 5). These findings indicate that ENST00000414355 expression is correlated with Cd exposure in Cd-exposed workers.

### ENST00000414355 expression was correlated with DNA damage of blood cells in Cd-exposed workers

A significant correlation was found between blood ENST00000414355 expression and DNA damage (r = 0.737, P = 0.002). The blood ENST00000414355 expression was moderately related to mean tail moment (TM) of 50 comets (r = 0.716, P = 0.003). The partial correlation (excluding UCd and BCd) analysis also found significant relationship between blood ENST00000414355 expression with TM, and DNA damage rate (control UCd: r = 0.676, P = 0.004; control BCd: r = 0.721, P = 0.001).

### ENST00000414355 expression was correlated with target gene expression in Cd-exposed workers

There was a significant positive correlation between ENST00000414355 expression and mRNA expression of ATM, ATR and ATRIP, while there was a significant negative correlation between ENST00000414355 expression and mRNA expression of DDB1,DDB2, OGG1, ERCC1, MSH2, RAD50 and XRCC1 in Cd-exposed workers. In addition, the associations between ENST00000414355 expression and target gene expression were further evaluated after adjustment for urine Cd concentration and blood Cd concentration that might affect ENST00000414355 expression and target gene expression, respectively, in Cd-exposed workers. A significant correlation between ENST00000414355 expression and target gene expression was still observed (Table 4). These findings indicate that ENST00000414355 expression correlate very well with target gene expression in Cd-exposed workers.

## Discussion

Increasing evidence confirms that lncRNAs have important biological functions and are associated with the progression of a variety of diseases. LncRNAs are becoming novel potential molecular markers for the disease diagnosis, treatment and prognosis^33^. LncRNAs are shown to be involved in the regulation of expression of genes encoding proteins. Moreover, lncRNAs may regulate the DNA damage and DNA repair at epigenetic, transcription and post-transcription levels^34^,^35^.

Cadmium is an important heavy metal widely used in nickel-cadmium batteries, metal plating, pigments, plastics, and alloys^36^. It may stimulate the free-radical production, resulting in oxidative deterioration of lipids, proteins and DNA, and initiating various pathological processes in humans and animals^37^. Several reports have shown that cadmium can induce DNA damage^38^,^39^. However, the underlying mechanism remains to be elucidated, and few reports have shown the effects and toxic mechanism of cadmium on the respiratory system. No study has been conducted to investigate the roles of lncRNAs in cadmium toxicity. In our previous study, results showed Cd could increase DNA damage and decrease DNA repair capacity^40^.

In the present study, the lncRNA expression profile was detected in Cd-induced 35^th^ cells by microarray assay. Of differentially expressed lncRNAs, 369 lncRNAs had up-regulated expression in the liver, whereas 90 had down-regulated expression in Cd-induced 35^th^ cells. In addition, the expression of 10 of the most significantly up-regulated lncRNAs in Cd-induced 35^th^ cells was further validated by real time PCR. To our knowledge, this was the first report regarding the potential role of lncRNAs in cadmium toxicity and Cd-induced carcinogenesis.

Among the 10 lncRNAs in Cd-induced 35^th^ cells, lncRNA-ENST00000414355 exhibited the highest expression as shown in qPCR. The GeneSymbol of lncRNA-ENST00000414355 is CR848007.6, which located at chromosome 9 and its size is 149891 bp. It is an intergenic lncRNA, and is 44021071 bp at 3′ end of gene encoding apoptosis inhibitor 5, and 44021414 bp at 5′ end of gene encoding leucine-rich repeat-containing protein 4C precursor. GO-pathway analysis showed the up-regulated ENST00000414355 mainly involved the following pathways: DNA damage and repair, biological cycle, etc. There were 22 potential target mRNAs regulated by ENST00000414355. Most of the targets mentioned here have been reported to be linked to cancers. These findings suggest that lncRNA-ENST00000414355 plays an important role in cadmium toxicity and Cd-induced carcinogenesis. Moreover, ENST00000446135, ENST00000451446 and uc004bxy.1 also showed relatively over five folds higher expression in Cd-induced 35^th^ cells compared with untreated 16HBE cells. They may also play roles in cadmium toxicity. Therefore, We will focus on these LncRNAs in our future research to confirm their functions and explore the significance in cadmium toxicity.

To verify the role of ENST00000414355 in cadmium toxicity, the expression of ENST00000414355 in untreated 16HBE cells and Cd-induced 35^th^ cells was knocked down via small interfering RNA. Results showed siRNA ENST00000414355 significantly inhibited the growth of DNA-damaged 16HBE cells during the Cd-induced malignant transformation. Moreover, ENST00000414355 knockdown also decreased the mRNA expression of DNA damage related genes ATM, ATR and ATRIP, but increased that of DNA repair related genes in Cd-induced 35^th^ cells. These findings suggested that siRNA ENST00000414355 activated DNA repair signaling pathway in Cd-induced carcinogenesis. Then, the RNA-protein interaction of lncRNA and corresponding TFS was further analyzed based on the Chip-Seq database. Results showed that there was highly enriched region between ENST00000414355 and DDB1, DDB2 and OGG1. The prediction of TFBS and further catRAPID analysis indicated that Polymerase 2 (Pol2) was a RNA-protein interaction between ENST00000414355 and DDB1, DDB2 and OGG1, which may be a potential mechanism of ENST00000414355 modulating the expression of DNA repair related genes DDB1, DDB2 and OGG1 in cadmium toxicity and Cd-induced carcinogenesis by the NAT (Natural Antisense Transcripts) theory^41^,^42^.

Many cellular and molecular events are involved in the toxic effects of chemical carcinogens^43^,^44^,^45^,^46^, but no study has been conducted to investigate lncRNAs as new biomarkers of Cd exposure. The present study was undertaken to investigate the role of lncRNAs in Cd toxicity of animal model and Cd-exposed workers. The animal model of sub-chronic Cd exposure used in this study was established by continuous intra-peritoneal injection of CdCl_2_ for 14 weeks. The cadmium toxicity was evaluated by the weight coefficient, histo-pathological examination and liver and renal function (ALT, AST, SCR, BUN and 24-h Pro) detection. The metal concentration of the blood reflects the recent exposure, and that of the urine reflects the body burden after a long-term exposure, while that of tissues reflects the metal accumulation and organ damage^47^,^48^,^49^. In the present study, the expression of lncRNA-ENST00000414355 in the lung of Cd-treated rats was positively correlated with the Cd exposure and the degree of organ damage, suggesting that lncRNA-ENST00000414355 reflects the accumulation of cadmium in the body and the organ damage. ENST00000414355 expression in the body is useful in predicting the Cd-induced toxicity.

In addition, the expression of lncRNA-ENST00000414355 was also detected in the blood and urine of workers exposed to Cd. Results showed a strong positive correlation between blood lncRNA-ENST00000414355 and urine Cd, DNA damage, and expression of target genes, suggesting that blood lncRNA-ENST00000414355 is potentially a novel biomarker of Cd-exposure in humans^47^. Subjects with urine Cd exhibited significantly higher blood lncRNA-ENST00000414355 expression than those without urine Cd, suggesting that, even at a lower range of urine Cd concentration, the change in blood lncRNA-ENST00000414355 may reflect the alteration in Cd accumulation.

Taken together, our study for the first time determines the genome-wide lncRNAs expression profile in the Cd-induced malignant transformation by microarray assay. Our results display that some lncRNAs were aberrantly expressed in CdCl_2_ treated cells when compared with untreated 16HBE cells. In addition, lncRNA-ENST00000414355 may serve as a partial or key signature of DNA repair related epigenetic mechanisms underlying the cadmium toxicity. LncRNA-ENST00000414355 is a novel valuable biomarker of cadmium exposure and cadmium toxicity and may become a significant biomarker for field investigations and risk assessment in humans exposed to occupational and environmental cadmium.

## Materials and Methods

### Cell culture and treatments

16HBE cells were morphologically transformed using CdCl_2,_ as previously described (Lei *et al.*, 2008)^31^. Untransformed 16HBE cells (controls); Cd-transformed cells at the 5^th^ (5 μmol L^–1^ Cd for 2 weeks), 15^th^ (5 μmol L^–1^ Cd for 6 weeks), and 35^th^ (5 μmol L^–1^ Cd for 14 weeks) passage were cultured in RPMI-1640 containing L-glutamine, 10% fetal bovine serum (FBS) and 1% penicillin/streptomycin (Life Technologies) at 37 °C in a humidified atmosphere with 5% CO_2_. The cells were passaged twice weekly and cells in logarithmic growth phase (2–5 × 10^5^ cells/mL) were harvested for following experiments.

### Animals and Cadmium Exposure^32^

Specific-pathogen-free (SPF) Sprague-Dawley (SD) rats (90 ± 10 g) were purchased from the Guangdong Medical Laboratory Animal Center (Licence No. SCXK 2008-0002, Guangdong, China) and housed under pathogen-free conditions in Laboratory Animal Center of Guangzhou Army General Hospital [Licence No. SYXK (Military) 2007-33, 2008C1230034834, Guangdong, China]. Ninety-six SD rats (half male and half female) were randomly divided into 4 groups. Rats were sub-chronically exposed to Cd by intra-peritoneal injection of CdCl_2_ in normal saline (Sigma, St. Louis, MO, USA) at different concentrations (high dose: 1.225 mg/kg; mid-dose: 0.612 mg/kg and low dose: 0.306 mg/kg). Rats in control group were intra-peritoneally injected with 0.5 mL of normal saline. Cd treatment was performed five times weekly. After 14 weeks, 24-h urine samples were collected. On the second day, rats were anesthetized and blood was collected from the heart and stored at 4 °C. The liver, kidney, heart and lung were harvested and stored in liquid nitrogen. The animal handling and experimental procedures were approved by the Animal Experimental Ethics Committee of Guangzhou Army General Hospital (Guangzhou, China).

### Study population

A total of 181 workers were recruited from a Cd refinery factory with the assistance of Center for Disease Control and Prevention, Institute for Health Supervision in Shenzhen, P.R. China. The workers included production workers, machine maintenance workers, product development personnel, management personnel and other personnel engaged in cleaning, service, security, and so on. Detailed information including the age, marital status, smoking habits, alcohol consumption, professional and medical history was collected from each subject and evaluated by well-trained interviewers. In addition, the workers were asked to receive a comprehensive physical examination. The physical examination included detection of blood pressure and pulse rate, examination of the throat and pharynx, detection of lung function, electrocardiography, liver and kidney ultrasonography, cardiopulmonary X-ray, and detection of blood cells, serum alanine aminotransferase (ALT), urinary Cd and creatinine (Cr). In this study, subjects who could not provide reliable information on the smoking history, had a smoking history or had a history of kidney or liver diseases were excluded. Finally, 181 non-smoking subjects (109 males and 72 females) with the age ranging from 23 to 50 years were included for analysis.

### Microarray Assay and Computational Analysis

RNA purified from total RNA after the removal of rRNA was amplified and transcribed into fluorescent cRNA along the entire length of the transcripts without 3′ bias using a random priming method. The cRNA was labeled and hybridized to the Human LncRNA Array v2.0 (8 × 60 K, Arraystar). In addition, 33,045 lncRNAs and 30,215 coding transcripts collected from the most authoritative databases, such as RefSeq, UCSC Knowngenes, Ensembl and many related literatures, were detected by microarray assay. The criteria were as follows: cut-off of fold-change: 2.0; positive value: up-regulation; negative value: down-regulation. Log fold-change means a log2 value of the absolute fold-change. The fold-change and P-value were calculated from the normalized expression. Arraystar LncRNA Array Protocol: Step1, Preparation of the RNA Sample, kit and reagents (TRIzol Reagent [Invitrogen life technologies], Biopulverizer [biospec], and Mini-Bead-Beater-16 [biospec]); Step 2, Total RNA Clean-up and RNA QC; Step 3, Preparation of labeling reaction; Step 4, Purification of the labeled/amplified RNA and labeled cRNA QC; Step 5, Hybridization; Step 6, Microarray Washing; Step 7, Scanning; Step 8, Extracting data using Agilent Feature Extraction Software. The microarrays were scanned using the Agilent Scanner G2505B, and the acquired array images were analyzed using the Agilent Feature Extraction software (version 11.0.1.1). Quantile normalization and subsequent data processing were performed using the GeneSpring GX v11.5.1 software package (Agilent Technologies). The microarray assay was performed by KangCheng Bio-tech, Shanghai P.R. China. The microarray data discussed in this paper have been deposited in NCBI Gene Expression Omnibus and are accessible with the GEO Series accession number GSE50783 (http://www.ncbi.nlm.nih.gov/geo/query/acc.cgi?acc=GSE50783).

### Gene ontology (GO) and pathway analysis

To investigate the function and associated pathways of differentially expressed mRNAs, GO and pathway analyses were performed. GO annotations of microarray genes were downloaded from NCBI (http://www.ncbi.nlm.nih.gov/), UniProt (http://www.uniprot.org/) and the Gene Ontology (http://www.geneontology.org/). The elim Fisher algorithm was used to perform a GO enrichment test and GO categories with P < 0.05 were reported^50^. Pathway annotations of microarray genes were downloaded from KEGG (http://www.genome.jp/kegg/) and a Fisher exact test was performed in order to locate the significant enrichment pathway. The resulting P values were adjusted using the Benjamini Hochberg false discovery rate (BH FDR) algorithm. Pathway categories with a FDR < 0.05 were reported.

### Construction of Coding-non-coding Gene Co-expression Network

The lncRNA-mRNA co-expression network was constructed based on the correlation between differentially expressed lncRNAs and mRNAs. (i) preprocessing of data: if one coding gene has different transcripts the median value was taken to represent the gene expression values, without special treatment of lncRNA expression values; ii) data were screened and the subset of data were removed according to the lists of the differential expression of lncRNA and mRNA obtained from the GO and pathway analyses; iii) the Pearson correlation coefficient (PCC) was calculated and the R value was used to calculate the correlation coefficient between lncRNA and coding genes; iv) Pearson’s correlation coefficient was used for screening; RNAs with a Pearson’s correlation coefficient of ≥0.99 were considered significant and the lncRNA-mRNA co-expression network was constructed by Cytoscape software (The Cytoscape Consortium, San Diego, CA, USA). The triangle nodes represented the lncRNA, and circular nodes represented the mRNA, where nodes with co-expression of more expressed genes have a more extensive relationship with the gene. The K-core indicates the gene expression.

### Quantitative real-time PCR

Total RNA was isolated using the TRIzol reagent. Reverse transcription was performed using a TITANIUM real-time PCR (RT-PCR) kit (Clontech, Mountain View, CA) according to the manufacturer’s instructions. The gene expression was quantified using a fluorescence-based RT-PCR according to manufacturer’s instructions (Bio-Rad Laboratories). The sequences of primers used for RT-PCR are shown in Table 5.

### Small interfering RNA (siRNA)

To inhibit lncRNA-ENST00000414355, 50 nM of siRNA (siRNA ENST00000414355-4311, siRNA ENST00000414355-4312, or siRNA ENST00000414355-4313; Shanghai Genepharma, China) were transfected into untreated 16HBE cells and Cd-transformed 35^th^ passage cells using Lipofectamine 2000 reagent according to the manufacturer’s instructions. Cells transfected with scramble-control siRNA (negative control) were used as controls. Cells were harvested at 72 h after transfection. When compared with control, only siRNA ENST00000414355-4311 and siRNA ENST00000414355-4312 successfully decrease the expression of LncRNA-ENST00000414355. The sequences of siRNA LncRNA-ENST00000414355 and scramble control siRNA are listed in Table 6.

### Comet assay

DNA damage was investigated using the comet assay^52^. 16HBE cells at different stages of Cd-induced malignant transformation, siRNA/ENST00000414355 transfected 35^th^ passage cells, negative control 35^th^ passage cells and white blood cells form Cd-exposed workers were diluted in PBS so that 3 or 4 cells could be observed in a single field at 400×. Comet assay was done according to the published protocol with a minor modification. Briefly, 100μL of low-melting agarose [1% (v/v) in PBS (pH 7.4)] at 37 °C was mixed with 10 μL of PBS containing lymphocytes and then transferred onto a pre-coated [0.5% (v/v) normal meltingag arose in PBS (pH 7.4)] slide. Electrophoresis was conducted for 20 min at 25 V. DNA damage was measured using an image analysis system (version 1.0, IMI Comet Analysis Software, China; ref.). Fifty cells were analyzed per slide, and the Olive tail moment (Olive TM) value was used as a measurement of DNA damage level as recommended.

### Bioinformatics analysis of lncRNA-ENST00000414355

To further analyze the LncRNA-ENST00000414355, the lncRNA-ENST00000414355-mRNA regulatory network was constructed. Pearson correlation coefficient and P-value of lncRNA-mRNA and mRNA-mRNA were calculated using FDR correction. LncRNA-ENST00000414355-mRNA regulatory network was drawn using the cytoscape (http://cytoscape.org/). The predicted target genes were input into the database Annotation Visualization and Intergrated Discovery (DAVID, http://david,abcc,ncifcrf,gov/). GO was used to identify the molecular function represented in the gene profile. In addition, the KEGG (Kyoto Encyclopedia of Genes and Genomes) database (http://www.genome, ad, jp/kegg) and BioCarta (http://www.biocarta,com) were used to analyze the roles of these target genes in the pathways.

In order to explore the potential targets of lncRNA-ENST00000414355, the RNA-protein interaction of lncRNA and corresponding TFs was analyzed based on the catRAPID algorithm, a free resource which can be obtained online (http://service.tartaglialab.com/page/cat rapid_group). The experimental determination of ribonucleoprotein (RNP) complexes is a slow and difficult process, and the number of experimentally determined structures of RNP complexes is still rather scarce. Thus, computational prediction of RNP complex structures would greatly facilitate the investigation of protein-RNA interactions and their molecular function. Through the calculation of secondary structures, hydrogen bonding and van der Waals contributions, catRAPID predicts protein-RNA interaction propensities with great accuracy (up to 89% on the ncRNA-protein interaction database, NPinter)^53^,^54^.

### Cadmium determination and organ functional and pathological examination of cadmium exposed rats

The cadmium level was determined using the cadmium standard solution (BZ/WJ/GB101/2009-1, Guangdong Occupational Health Inspection Center, Guangdong, China) by atomic absorption spectrometry (ZEENIT700, Analytik Jena, Jena, German). The concentration of urine cadmium was normalized by urinary creatinine (Cr). Tissue samples were fixed with 10% formalin and the pathological features were examined following the standard Hematoxylin and Eosin (HE) staining. The expression of ENST00000414355 and its target genes in the lung of low dose, mid-dose and high dose Cd exposed rats were measured using qPCR. Serum ALT and AST were used as biochemical markers of liver function. Blood urea nitrogen (BUN), serum creatinine (sCr) and 24-h Pro were used to evaluate the renal function. ALT, AST, BUN, SCR and 24-h Pro were measured using corresponding kits according to manufacturer’s instructions and automatic biochemistry analyzer (Hitachi 7600- 020/7170A: Tokyo, Japan). ALL animal experiments were performed in accordance with the principals of the Declaration of Helsinki. All experimental protocols were approved by Research Ethic Committee of Guangzhou Medical University.

### Collection and treatment of biological samples of the cadmium-exposed workers

Venous blood was collected after fasting for 10–12 h and transferred into anticoagulant and metal-free tube after 10–12 h of fasting to determine blood cadmium (BCd), blood routine examination, blood biochemical examination (ALT, AST, Cr and BUN), and detection of blood lncRNA-ENST00000414355 and its target genes. BCd concentrations were measured by atomic absorption spectrometry (ZEENIT700; Analytik Jena, Jena, Germany). Blood biochemistry was done with an automatic biochemical analyzer (HITACHI7600-020/7170A; Hitachi, Tokyo, Japan). The expression of lncRNA-ENST00000414355 and its target genes was measured by quantitative real-time PCR.

Urine samples were collected from all participants and transferred into a metal-free polyethylene bottle. These samples were diluted with equal volume of 0.3 mol/L HNO_3_ and stored at 4 °C untill further analysis. Cd level of the urine was measured by atomic absorption spectrometry (ZEENIT700; Analytik Jena, Jena, Germany). Cd standard curve was linear up to 25 μg/L and the detection limit was 0.33 μg/L. The internal standard of Cd was added to urine and analyzed, and a recovery rate of 98.2% was found.

All the procedures were performed in accordance with the principals of the Declaration of Helsinki. All experimental protocols of collecting and detecting the workers’ blood samples and urine samples were approved by Research Ethic Committee of Guangzhou Medical University. Informed consent was obtained from all subjects, and the personal information of samples involved in the study was not opened.

### Statistical analysis

Data are represented as rate (%) or mean ± standard deviation (SD; 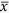 ± s) of three or more independent experiments. Statistical significance was determined using the chi square test for rate (%) in several independent experiments and Student’s t-test or analysis of variance (ANOVA) followed by Dunnett’s multiple comparison test for mean ± SD. The relationship between two groups was tested by Pearson or Spearman’s correlation analysis. Statistical analysis was performed with SPSS version 13.0 software. A value of *P* < 0.05 was considered statistically significant.

## Additional Information

**How to cite this article**: Zhou, Z. *et al.* Long non-coding RNAs as novel expression signatures modulate DNA damage and repair in cadmium toxicology. *Sci. Rep.* **5**, 15293; doi: 10.1038/srep15293 (2015).

## Supplementary Material

Supplementary Information

Table S1

Table S2

Table S3

Table S4

Table S5

Table S6

Table S7

## Figures and Tables

**Table 1:** DNA damage and its suppression during Cd-induced malignant transformation of 16HBE cells and siRNA/ENST00000414355 transfected 35^th^ passage cells determined by comet assay.

Cell type	DNA damage rate (%)	Tail length (μm)
Untransformed 16HBE cells	4.75	12.8 ± 1.76
5^th^ passage	10.45#	15.2 ± 3.54#
15^th^ passage	22.00*#	31.6 ± 2.80*#
35^th^ passage	46.75*	47.8 ± 2.36*
siRNA/ENST00000414355-4311 transfected 35^th^ passage cells	24.79*#	33.8 ± 4.43*#
siRNA/ENST00000414355-4312 transfected 35^th^ passage cells	23.98*#	32.4 ± 4.17*#
Negative control transfected 35^th^ passage cells	41.14*	44.56 ± 5.17*

The tail lengths of cells in which DNA damage was induced and suppressed were determined by comet assay in untransformed 16HBE cells, 5^th^, 15^th^, 35^th^ passage transformed cells, siRNA/ENST00000414355 transfected 35^th^ passage cells and Negative control transfected 35^th^ passage cells. *P<0.05, vs untransformed controls, ^#^P<0.05, vs 35^th^ passage cells.

**Table 2:** Correlation between lncRNA-ENST00000414355 expression and its target gene expression in Cd-exposed rats.

Target genes	Correlation coefficient (r)	P	Target genes	Correlation coefficient (r)	P
ATM	0.653	0.001	OGG1	−0.737	<0.0001
ATR	0.618	0.003	ERCC1	−0.531	<0.0001
ATRIP	0.631	<0.0001	MSH2	−0.529	0.002
DDB1	−0.701	<0.0001	RAD50	−0.604	<0.0001
DDB2	−0.685	<0.0001	XRCC1	−0.652	0.004

**Table 3:** Blood LncRNA-ENST00000414355 expression in Cd-exposed workers.

Exposure to Cd at different levels	N	Blood lncRNA-ENST00000414355 concentration	F	P
Ucd levels				
0–2 μg/g Cr	153	0.993 ± 1.416	49.485	<0.0001
2–5 μg/g Cr	20	2.647 ± 3.578		
>5 μg/g Cr	14	7.113 ± 5.467		
Urine β_2_-MG				
0–500 μg/g Cr	156	0.951 ± 1.720	70.329	<0.0001
500–1000 μg/g Cr	18	2.913 ± 1.383		
>1000 μg/g Cr	13	7.969 ± 5.204		
BCd level				
0–2 μg/l	130	0.918 ± 1.214	37.324	<0.0001
2–5 μg/l	40	2.023 ± 3.558		
>5 μg/l	17	6.126 ± 4.614		

**Table 4:** Correlation Analysis between lncRNA-ENST00000414355 expression and target gene expression in Cd-exposed workers.

Target genes	Correlation coefficient (r)	P	Target genes	Correlation coefficient (r)	P
ATM	0.514	<0.0001	OGG1	−0.719	<0.0001
ATR	0.671	0.002	ERCC1	−0.417	<0.0001
ATRIP	0.509	0.001	MSH2	−0.467	<0.0001
DDB1	−0.743	<0.0001	RAD50	−0.583	0.001
DDB2	−0.734	<0.0001	XRCC1	−0.617	0.017

**Table 5:** Primers used for Real-time PCR of selected lncRNAs and mRNA.

LncRNAs	Primers
ENST00000414355	Forward:5′−CAGAAAGAAGCCAAACAAGGAG−3′
	Reverse:5′− AACCACCAAACAGTCAGCAG−3′
ENST00000446135	Forward: 5′−GGGACAAGCAGCACAGAACT−3′
	Reverse:5′− CAGCAGAATAACGGCACAAG−3′
ENST00000451446	Forward: 5′−ATGATTGGCTCTTTCGCTGA−3′
	Reverse:5′− TCCACTTTCACAGGCATTTCT−3′
uc004bxy.1	Forward: 5′−GAAAACTGCCCCACATCATC−3′
	Reverse:5′− GTGCTCTCCTTTGACCCTGT−3′
uc003tcy.3	Forward: 5′−ATGGCGGTTTTGTCGAATAG−3′
	Reverse:5′− TGGACACAGCACATGTTTCA−3′
ENST00000439302	Forward: 5′−TCCTTCCTTGAAGCCTAGCA−3′
	Reverse:5′− TCAGCAGCAGCAGAAGATGT−3′
AK000930	Forward: 5′−CCAATGCAAGTGAACACGGG−3′
	Reverse:5′− TGGGATTTGCTGCATTTCACAG−3′
ENST00000436765	Forward: 5′−GAAGTCCCCAGAAACATCCA−3′
	Reverse:5′− CAGGCCTTGATGCCTTAGAC−3′
ENST00000457776	Forward: 5′−GTGTGTCCCCGAGAAAGTGT−3′
	Reverse:5′− TGCTAGGCTTCAAGGAAGGA−3′
AK021798	Forward: 5′−TTTTTCATGCCGACTGTCCCT−3′
	Reverse:5′−GCACACAAAACCTACAAAACCTC−3′
mRNAs	Primers
ATM	Forward:5´- TGCCAGACAGCCGTGACTTAC -3´
	Reverse: 5´- ACCTCCACCTGCTCATACACAAG -3´
ATR	Forward: 5′-GCCGTTCTCCAGGAATACAG-3′
	Reverse: 5′-GAGCAACCGAGCTTGAGAGT-3′
ATRIP	Forward: 5′-CAGCTGGAGACAGAGATCAA-3′
	Reverse: 5′-GACATTCCAGCCAAGGTACT-3′
DDB1	Forward: 5′-TGGTTGCCAAGCACCTACTA-3′
	Reverse: 5′-ACTGCGATCACCATGGAAGC-3′
DDB2	Forward: 5′-ATCCTGTCAACGCAGCTTGT-3′
	Reverse: 5′-GATGCCAGGCTGCCTTGAT-3′
OGG1	Forward: 5′- CCGAGCCATCCTGGAAGAAC-3′
	Reverse: 5′-CCATCAGGCAGATGCAGTCA-3′
ERCC1	Forward: 5′-CTTGTCCAGGTGGATGTGAA-3′
	Reverse: 5′-GCCTTGTAGGTCTCCAGGTA-3′
MSH2	Forward: 5′-CATCCAGGCATGCTTGTGTTGA-3′
	Reverse: 5′- GCAGTCCACAATGGACACTTC-3′
RAD50	Forward: 5′-GGGTTTCCAAGGCTGTGCTAA -3′
	Reverse: 5′-TCTGACGTACCTGCCGAAGT-3′
XRCC1	Forward: 5′-CAGCCGGATCAACAAGACAT-3′
	Reverse: 5′-CTGAGGAGGCAGCACTAGAA-3′
β-Actin	Forward: 5′-ACAGAGCCTCGCCTTTGCCGAT-3′
	Reverse: 5′-CTTGCACATGCCGGAGCCGTT -3′

**Table 6:** Sequences of lncRNA-ENST00000414355 siRNA and scramble control siRNA.

Name	lncRNA	Sequence(5′to 3′)
siRNA-4311	ENST414355-sense	5′-AGAAGCCAAACAAGGAGCUTT-3′
siRNA-4311	ENST414355-antisense	5′-AGCUCCUUGUUUGGCUUCUTT-3′
siRNA-4312	ENST414355-sense	5′-CCUAGGCACAGAUGCUAAUTT-3′
siRNA-4312	ENST414355-antisense	5′-AUUAGCAUCUGUGCCUAGGTT-3′
siRNA-4313	ENST414355-sense	5′-GGAGCUUUCUGCAGAAUGATT-3′
siRNA-4313	ENST414355-antisense	5′-UCAUUCUGCAGAAAGCUCCTT-3′
siRNA-NC	NC-sense	5′-UUCUCCGAACGUGUCACGUTT-3′
siRNA-NC	NC-antisense	5′-ACGUGACACGUUCGGAGAATT-3′
