# Supplementary material for: Long non-coding RNAs as novel expression signatures modulate DNA damage and repair in cadmium toxicology
Source: Sci Rep. 2015 Oct 16;5:15293. doi: 10.1038/srep15293 (PMC4607885; doi:10.1038/srep15293)
Supplement: Supplementary Information [file srep15293-s1.pdf]

Supplementary information

Long non-coding RNAs as novel expression signatures modulate DNA damage and repair in cadmium toxicology

Zhiheng Zhou[1], Haibai Liu[1], Caixia Wang[2], Qian Lu[3], Qinhai Huang[1], Chanjiao Zheng[1], Yixiong Lei[1]

1. School of Public Health, Guangzhou Medical University, Guangzhou 510182, People's Republic of China
2. Department of Internal Medicine of Guangzhou First People's Hospital, Guangzhou Medical University, Guangzhou 510180, P.R. China
3. Shenzhen Longgang District Center for Disease Control & Prevention, Shenzhen 518108, P.R. China

Correspondence and requests for materials should be addressed to Yixiong Lei (email: gz-leizeng@163.com)

Supplementary Figure 1

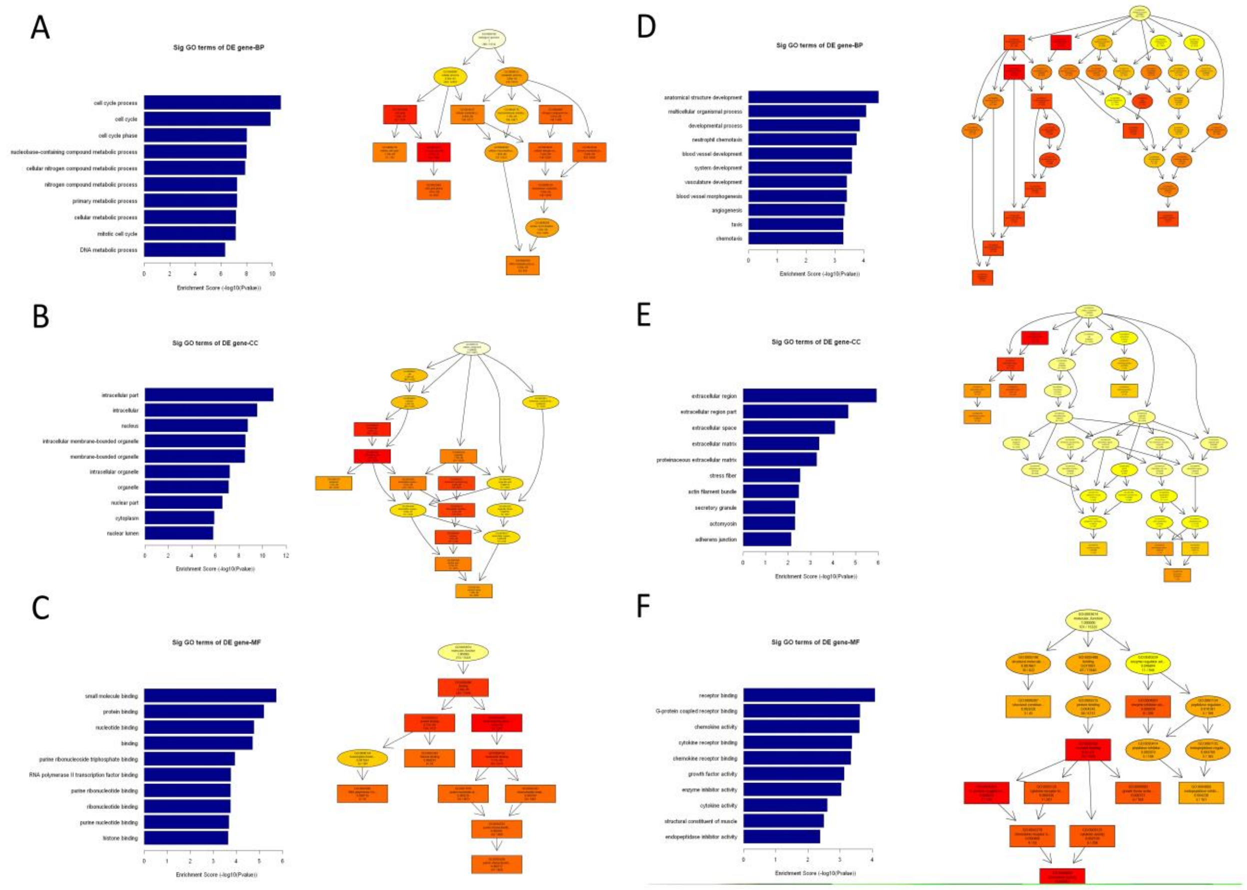

Figure.S1. GO analysis of differentially Expressed mRNAs

The top 10 of the most related parts were shown A-F; A-C showed biological process (A), cellular component (B) and molecular function (C) of up-regulated mRNAs, respectively. D-F showed biological process (D), cellular component (E) and molecular function (F) of down-regulated mRNAs.

Supplementary Figure 2

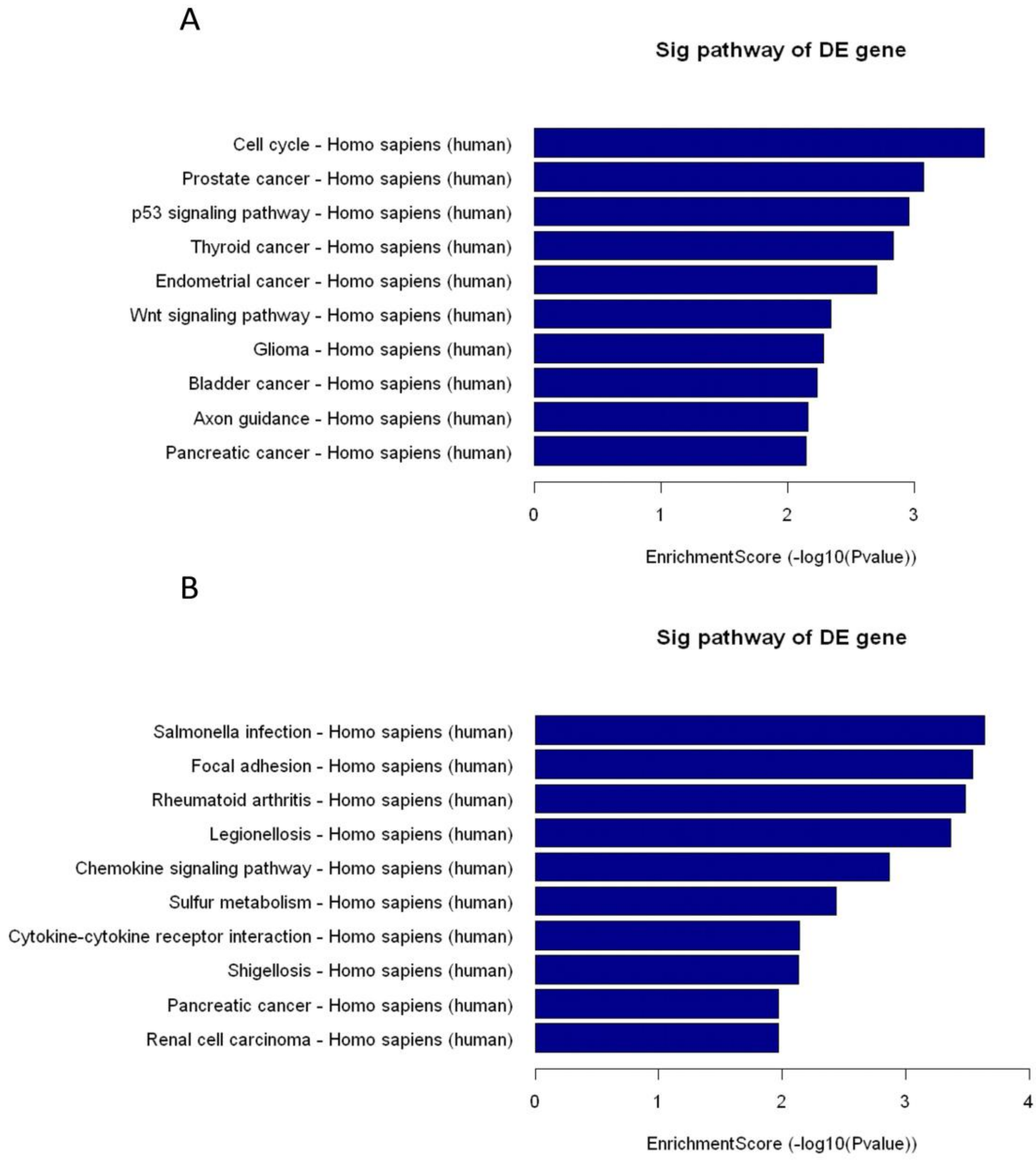

Figure.S2. Pathway analysis of differentially Expressed mRNAs

Pathways with P<0.05 were shown in figure. All the P values were transformed into log p value.

Supplementary tables

Table S1. LncRNA Expression Profiling Data. (XLS)

Table S2. Differentially Expressed LncRNAs. (XLS)

Table S3. mRNA Expression Profiling Data . (XLS)

Table S4. Differentially Expressed mRNAs. (XLS)

Table S5. GO analysis of differentially Expressed mRNAs. (XLS)

Table S6. Pathway analysis of differentially Expressed mRNAs. (XLS)

Table S7. GO and Pathway analysis of lncRNA-ENST00000414355. (XLS)
